# Context-dependent T-BOX transcription factor family: from biology to targeted therapy

**DOI:** 10.1186/s12964-024-01719-2

**Published:** 2024-07-04

**Authors:** Siwen Li, Xiangyuan Luo, Mengyu Sun, Yijun Wang, Zerui Zhang, Junqing Jiang, Dian Hu, Jiaqian Zhang, Zhangfan Wu, Yufei Wang, Wenjie Huang, Limin Xia

**Affiliations:** 1 Department of Gastroenterology, Institute of Liver and Gastrointestinal Diseases, Hubei Key Laboratory of Hepato-Pancreato-Biliary Diseases, Tongji Hospital of Tongji Medical College, Huazhong University of Science and Technology, Wuhan, Hubei Province 430030 China; 2 Hubei Key Laboratory of Hepato-Pancreato-Biliary Diseases, Hepatic Surgery Center, Tongji Hospital, Tongji Medical College, Clinical Medicine Research Center for Hepatic Surgery of Hubei Province, Key Laboratory of Organ Transplantation, Huazhong University of Science and Technology, Ministry of Education and Ministry of Public Health, Wuhan, Hubei 430030 China; 3 State Key Laboratory of Holistic Integrative Management of Gastrointestinal Cancers, National Clinical Research Center for Digestive Diseases, Xijing Hospital of Digestive Diseases, Fourth Military Medical University, Xi’an, 710032 China

## Abstract

T-BOX factors belong to an evolutionarily conserved family of transcription factors. T-BOX factors not only play key roles in growth and development but are also involved in immunity, cancer initiation, and progression. Moreover, the same T-BOX molecule exhibits different or even opposite effects in various developmental processes and tumor microenvironments. Understanding the multiple roles of context-dependent T-BOX factors in malignancies is vital for uncovering the potential of T-BOX-targeted cancer therapy. We summarize the physiological roles of T-BOX factors in different developmental processes and their pathological roles observed when their expression is dysregulated. We also discuss their regulatory roles in tumor immune microenvironment (TIME) and the newly arising questions that remain unresolved. This review will help in systematically and comprehensively understanding the vital role of the T-BOX transcription factor family in tumor physiology, pathology, and immunity. The intention is to provide valuable information to support the development of T-BOX-targeted therapy.

## Introduction

T-BOX transcription factors are evolutionarily highly conserved and are characterized by a conserved DNA-binding domain termed T-BOX, identifying and specifically binding to its target DNA domain. TBXT, also known as Brachyury (meaning short tail), was first described in 1927 by the scientist Dobrovolskaia-Zavadskaia in a mutant mouse strain. The *TBXT* mutation in mouse results in a shorter tail or complete absence of the tails [1]. Since then, many T-BOX transcription factors have been uncovered, and their essential functions in physiological and pathological conditions have gradually been revealed. Physiologically, T-BOX factors are involved in many developmental processes, including embryonic, organogenic, and limb development [2]. However, if dysregulation of T-BOX factors occurs, normal body function may be disrupted, and the individual becomes vulnerable to several diseases, including cancer. For example, *TBX1* and *TBX5* haploid insufficiency respectively causes DiGeorge syndrome charactered as pharyngeal, cardiac, thymic, craniofacial hypoplasia and Holt–Oram syndrome (HOS) charactered as cardiovascular malformations, skeletal malformations [2, 3]. Overexpression of TBXT, TBX15, and TBX19 causes tumor invasion, migration, and metastasis [4–6]. Moreover, TBXT and TBX15 are associated with antitumor drug resistance [7–9].

Numerous T-BOX factors have been found to be dysregulated in specific tumors. Due to the vital roles of T-BOX factors in processes such as cell proliferation and organogenesis, aberrant expression is likely to be involved in tumor initiation and progression. Moreover, the same T-BOX molecules play differential roles when involved in different tumor processes. T-BOX factors show great potential as targets or biomarkers for tumor therapy. However, enormous challenges remain in realizing the clinical translation of T-BOX factor targeting.

This review summarizes the information on the role of T-BOX factors in physiological, immunological, and tumor-related pathways. Specifically, we have made certain discussions about the different context-dependent roles of the same T-BOX molecule.

## General overview of the T-BOX family

**Fig. 1:**
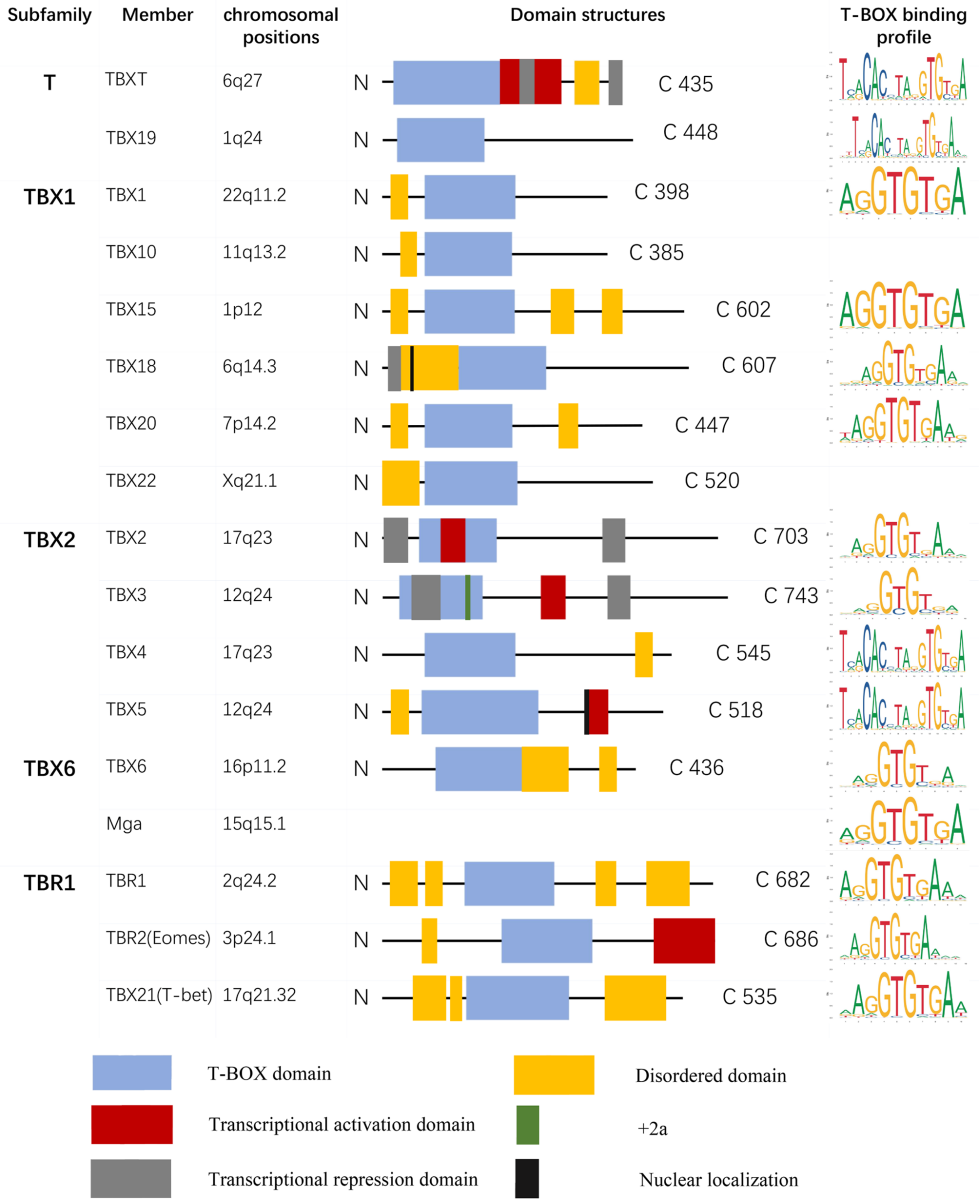
Domain structure and binding profile of human T-BOX transcription factors. T-BOX family members are classified into five subfamilies and have the same T-BOX domain sequence, which is an evolutionarily conserved DNA-binding motif and a representative signature of the T-BOX family. In addition to the shared T-BOX domain, T-BOX members have specific structural domains that result in functional differences. The diagram shows the DNA-binding domain (T-BOX, blue box), inhibitory domains (grey box), activation domain (red box), +2a splicing variant (green box), and nuclear localization sequence (black box). The chromosomal positions and the domain structures of T-BOX members have been ascertained. The predicted T-BOX binding profiles were obtained from the JASPAR public database

The family of *T-BOX* genes existing in all metazoans has ancient origins and exhibits involvement in a wide range of processes related to growth and development [2, 10]. These genes have been demonstrated to act as transcriptional activators or repressors. T-BOX members share a common protein motif with TBXT, spanning 180–200 amino acid residues, which binds to DNA in a specific way [10]. Currently, 17 T-BOX transcription factors have been identified in humans. The relationship between *T-BOX* genes was reflected by the homology of DNA-binding domains, and five subfamilies were identified: T (TBXT and TBX19), TBX1 (TBX1, TBX10, TBX15, TBX18, TBX20, and TBX22), TBX2 (TBX2, TBX3, TBX4 and TBX5), TBX6 (TBX6 and MGA), and TBR1 (TBR1, EOMES, and T-BET) [2] (Fig. 1). Owing to their common and unique structural characteristics, T-BOX factors play a crucial role in various aspects of development.

### Physiological functions of T-BOX genes

The T-BOX transcription factor family members play various roles in several developmental processes. A single *T-BOX* gene can be involved in multiple developmental processes and a single developmental process can involve multiple *T-BOX* genes [2]. Dysregulation of T-BOX expression in tissues during development can lead to multiple human syndromes (Table 1) Increasing evidence suggests that the T-BOX family serves as a promising biomarker associated with the diagnosis, identification of therapeutic targets and prognosis of various cancers. Familiarity with the role of the T-BOX family in physiology is important to gain insights into the mechanisms of T-BOX dysregulation especially in tumors.

**Table 1:** The roles of T-BOX members in developmental processes and human syndromes

Subfamily	Members	Transcriptional regulatory effects	Role in development	Developmental effect of mutation in mice	Human syndromes	Reference
T	TBXT (Brachyury)	Transcriptional activation	Embryonic development Cardiogenesis Limb development Mesoderm development Stem cell	Defects in notochord, allantois, and primitive streak as well as dysplasia of urinary bladder	Spinal cord defects	[11, 12, 170, 171]
	TBX19	Transcriptional activation	Pituitary gland development	Abnormal terminal differentiation of proopiomelanocortin (POMC)-expressing cells in the pituitary gland. Adrenocorticotropic hormone (ACTH) deficiency and adrenal, gonadal, and pigment defects	Recessive isolated ACTH deficiency (IAD)	[2, 172, 173]
TBX1	TBX1	Transcriptional activation	Cardiogenesis Limb development Craniofacial effects	Pharyngeal, cardiac, thymic, and craniofacial hypoplasia	DiGeorge syndrome	[2, 26]
	TBX15	Transcriptional activation	Limb development Craniofacial effects	Craniofacial and limb malformations and pigment pattern alterations	Cousin syndrome	[2, 172]
	TBX18	Transcriptional activation	Embryonic development Cardiogenesis Limb development Urinary system	Abnormal somatic polarization, hydroureter, and hydronephrosis		[16, 34, 35]
	TBX20	Transcriptional activation	Cardiogenesis	Defects in chamber septation, chamber growth, endocardial pads, valvular production, and abnormal action potentials	Congenital heart defects, dilated cardiomyopathy, and arrhythmias	[21, 23]
	TBX22	Transcriptional activation	Craniofacial effects	Cleft palate and tongue tonicity	Cleft palate and ankyloglossia	[29]
TBX2	TBX2	Transcriptional inhibition	Cardiogenesis Limb development Craniofacial effects Cell cycle	Cleft palate	X-linked cleft palate with ankyloglossia (CPX)	[28, 31, 174]
	TBX3	Transcriptional inhibition	Embryonic development Cardiogenesis Limb development Craniofacial effects Stem cell	Defects in upper limb, areola, dental structure, heart, and genitals and cleft palate	Ulnar-mammary syndrome	[28, 30, 49, 175, 176]
	TBX4	Transcriptional activation	Embryonic development Limb development Respiratory system	Defects in growth and angiogenesis of allantois, patellar aplasia or hypoplasia, and abnormalities of pelvis and foot	Small patella syndrome (SPS) and pulmonary hypertension	[24, 25, 32]
	TBX5	Transcriptional activation	Cardiogenesis Limb development Nervous system	Congenital conduction disorders, early-onset atrial fibrillation, and abnormal diastolic function of heart	Holt–Oram syndrome (HOS) and congenital heart disease (CHD)	[20, 33, 172]
TBX6	TBX6	Transcriptional activation	Embryonic development Cardiogenesis	Hypoplasia of posterior somite	Spondylocostal dysostosis	[15]
TBR1	TBR1	Transcriptional activation	Nervous system	Neurological disorders	Autism spectrum disorder (ASD)	[2, 36]
	TBR2 (EOMES)	Transcriptional activation	Embryonic development Limb development	Embryo stagnation at blastocyst stage and loss of trophoblast function	Microcephaly syndrome	[2, 17]
	TBX21 (T-BET)	Transcriptional inhibition		Airway hyperresponsiveness and absence of T helper - type 1(Th1) cells	Asthma susceptibility and immune hepatic injury	[2, 177]

#### Embryonic development

T-BOX factors are widespread in early embryonic tissues and play indispensable roles in embryo development [3]. TBXT is expressed in primitive streak of the early embryo, which is responsible for posterior mesoderm development. In the absence of TBXT, mouse embryos exhibit significant defects in the development of notochord, allantois, and primitive streak [11, 12]. TBX4 is expressed in the allantois and promotes normal development. *Tbx4*-null mouse embryos show inability to produce the biomarkers of allantois differentiation, resulting in the death of these embryos due to the inability to establish umbilical cord connections [13, 14]. TBX6 is expressed in the paraxial mesoderm and is significant associated with somite development [15]. In mice, TBX18 acts downstream of Mesp2 and Delta/Notch signaling to maintain somatic compartmentalization by preventing anterior or posterior cells from migrating to the adjacent compartments [16]. EOMES is required for the growth and differentiation of trophoblast cells and is significantly upregulated in the trophoblast ectoderm [17].

#### Cardiogenesis

TBXT, TBX1, TBX2, TBX3, TBX5, TBX6, TBX18, and TBX20 are associated with all processes of cardiac development, including the development of inflow tract, outflow tract, individual ventricular chambers, heart valves, septa, and the differentiation of aortic arch and cardiac conduction system [2]. *TBX5* haploinsufficiency can lead to congenital conduction disorders. *TBX5* mistranslation variants cause cardiac electrophysiological abnormalities resulting in early-onset atrial fibrillation [18, 19]. TBX5 deficiency leads to reduced levels of sarco(endo)plasmic reticulum Ca^2+^-ATPase isoform 2a, resulting in abnormal diastolic function of the heart [20]. TBX20 exists a fundamental role in cardiovascular development, and its mutations are broadly involved in human congenital heart defects (CHDs), including defects in chamber septation, chamber growth, and valvular production. Additionally, molecular variants of TBX20 are associated with dilated cardiomyopathy and arrhythmias [21, 22]. Human *TBX20* mutations reduce the key channel Kv11.1 responsible for the ventricular repolarizing current and inward rectifying currents, leading to abnormal action potentials [23].

#### Limb development and craniofacial effects

*T-BOX* genes, including *T*, *Tbx1*, *Tbx15*, *Tbx18*, *Eomes*, and the *Tbx2* subfamily members, are involved in limb development [2]. *T-BOX* genes exist in the somatic region prior to the limb budding stage and are expressed until the late limb developmental stage. Mutations in several *T-BOX* genes can cause human diseases (Table 1). Small patella syndrome, characterized by patellar hypoplasia and abnormalities of the pelvis and the foot, is caused by LMX1B and TBX4 mutations in the PITX1/TBX4 signaling cascade [24, 25].

Several T-BOX factors significantly affect craniofacial development. TBX1 regulates the expression of pharyngeal growth factors and plays a vital role in craniofacial muscle development [26, 27]. *Tbx2* and *Tbx3* are regulated by bone morphogenetic protein signals and are expressed explicitly during palatal shelf development, where they play a crucial role. Double heterozygous *Tbx2-* and *Tbx3*-mutated mice and homozygous mutants of *Tbx2* mice exhibit a cleft palate phenotype [28]. TBX22 is also necessary for palate development, and its mutation leads to X-linked cleft palate and tongue tonicity [29].

#### Other functions

The importance of the T-BOX factors for the development of the organism is also reflected in other ways, such as their functions in stem cell development, cell cycle, and urinary, visual, respiratory, and nervous systems [28, 30–36]. Specifically, TBX3 is a major transcription factors associated with the regulatory circuitry of the pluripotent state. It maintains the pluripotent state of embryonic stem cells in vitro, enhances cell self-renewal, and suppresses differentiation [30]. TBX2 functions as a transcriptional repressor. TBX2 promotes the process of cells bypass senescence by downregulating the expression of the negative cell cycle regulator p21 [31]. This property of TBX2 and TBX3 makes it to be an important regulator during the tumor process. Once TBX2 or TBX3 expression is dysregulated, it may be involved in the formation of tumor stemness. TBX4 plays a key role in lung branch morphogenesis. The monoallelic-pathogenic variant of *TBX4* is the second most prevalent hereditary cause of pulmonary hypertension [32]. TBX5 is expressed during upward-preferring ON direction-selective ganglion cell (up-oDSGC) development in mice, and regulates vertical image stabilization [33]. TBX18-positive renal mesenchymal cells are necessary for urinary system development. Neonatal *Tbx18–/–* mice exhibit a distinct hydroureteric and hydronephrotic phenotype [34, 35]. Furthermore, TBR1 plays a crucial role in brain development by mediating neuronal migration, fate specification, axonal bundle formation. Mutations in the *TBR1* gene or deletion of the chromosome it resides on can lead to neurological disorders [2, 36].

With the development of histological analysis in recent years, intracellular molecular mechanisms have been increasingly revealed. A recent study comparing the gene expression patterns of trophoblasts at different developmental stages using single-nucleus RNA sequencing revealed the spatial heterogeneity of TBX3 during trophoblast development. TBX3 was significantly up-regulated during the transition from chorionic cell trophoblast to syncytial trophoblast, but down-regulated as the chorionic cell trophoblast progressed to extrachorionic trophoblast [37]. This implies that TBX3 may indeed be a key regulator in the development of the human trophoblast lineage. Not only in embryonic trophoblast development, the important roles of the T-BOX family in various developmental processes have been revealed. However, the segregation and integration of T-BOX molecular transcriptional networks during life development remain unclear. With technological updates and the development of the scientific community, the role of the T-BOX family in human development can be elaborated in more comprehensive detail by utilizing emerging histological techniques.

### Dysregulation of T-BOX factors in cancers

**Fig. 2:**
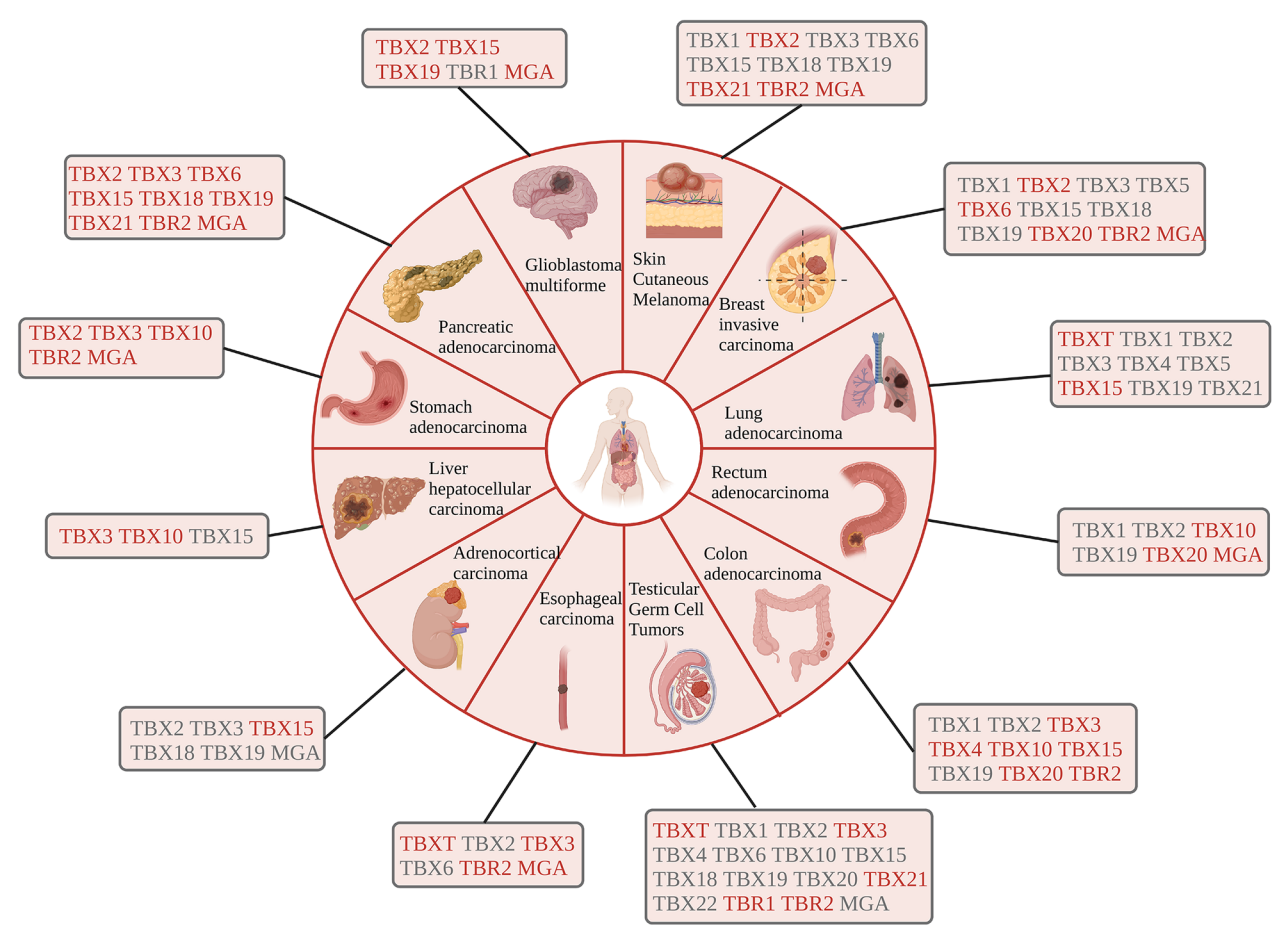
Dysregulation of T-BOX genes in pan-cancers. Overview of expression of T-BOX family members in pan-cancers. The regulation of the T-BOX family in different types of cancers is indicated in this figure. Red font indicates that the T-BOX member is up-regulated in the cancerous tissue compared to that in the corresponding para-cancerous tissue; conversely, grey font indicates that the T-BOX member is down-regulated in the cancerous tissue compared to that in the corresponding para-cancerous tissue. Data were analyzed using the GEPIA software (Gene Expression Profiling Interactive Analysis; cancer-pku.cn), which helps in analysis of the expression levels of T-BOX mRNAs

Given that T-BOX plays a crucial role, particularly in organogenesis and embryonic development, its dysregulation could easily lead to disruptions in cellular development, ultimately progressing toward carcinogenesis. Several studies have revealed that the abnormal expression of T-BOX factors is related to tumor initiation and progression. For example, TBXT is highly expressed in several human tumor tissues and cell lines such as adenoid cystic carcinoma (ACC), breast cancer, squamous cell carcinoma (SCC), non-small cell lung cancer (NSCLC), and prostate cancer (PCa) [4, 38–43], and acts as a controller of epithelial-mesenchymal transition (EMT). TBXT is not overexpressed in normal tissues. It is worth noting that TBXT is expressed at low levels in testes, spleen, cluster of differentiation 19^+^ (CD19^+^) (resting) lymphocytes, and thyroid [7, 44]. TBX2 is highly expressed in a variety of tumors, such as hepatocellular carcinoma (HCC), gastric cancer (GC), glioblastoma (GBM), and breast cancer [45–48]. In adult tissues, TBX3 is mainly expressed in cancers of epithelial or mesenchymal origin [49], such as head and neck SCC, GC, ovarian cancer, cervical cancer (CC), pancreatic cancer, bladder cancer, and HCC [30, 50, 51]. Here, we have summarized the expression of *T-BOX* genes at the mRNA level in pan-cancers (Fig. 2), and their specific mechanisms of action in cancer are described in detail in later sections.

#### T-BOX transcription factors associated with tumors: clinicopathological characteristics

Multiple T-BOX molecules, including TBXT, TBX1, TBX2, TBX5, TBX15, TBX19, and T-BET, are associated with clinicopathological features. The T-BOX family has potential value as clinical biomarkers. Specifically, overexpression of TBXT is clinic characteristic of spinal cord tumors [52]. However, TBXT plays a tumor-suppressive biomarker in gliomas. Low expression of TBXT is an independent biomarker of poor prognosis in gliomas [53]. TBXT plays completely opposite roles in different tumors, which may be due to the differences in the microenvironment of the organ where the tumor is located. Differences in various infiltrating factors such as cytokines and immune cells in the microenvironment lead to inconsistent clinic effects of TBXT. However, the specific mechanisms behind these phenomena still require further exploration. High expression of TBX1 in PCa is positively correlated with poor tumor pathological staging, poor pathological lymph node staging, and short progression-free survival [54]. Previous studies have found that GC and breast tumors patients with high TBX2 expression levels exhibit a worse prognosis than those with low TBX2 expression [48, 55]. The level of TBX15 in patients with clear cell renal cell carcinoma (ccRCC) correlates with overall and disease-free survival rates [6]. TBX15 is associated with poor clinical pathological features and worse survival prognosis in GBM patients [56]. It can serve as a predictive factor for prognosis in GBM patients. Elevated expression levels of TBX19 levels indicate poor prognosis of patients with HCC [5]. In colorectal cancer, the inhibition of TBX5 expression is controlled by promoter methylation and is associated with poor prognosis [57].

However, some T-BOX molecules play a protective role in tumors and are associated with favorable patient outcomes. In particular, T-BET, as an important transcription factor related to immune function, plays a key role in anti-tumor. Carbohydrate antigen 125 (CA125) is a prognostic and diagnostic marker for ovarian cancer [58]. The density of T-BET-positive infiltrating lymphocytes in tumor nests is significantly correlated with serum CA125 levels and distant metastases, suggesting the higher infiltrating T-BET-positive lymphocytes density in the human epithelial ovarian tumor tissue, the higher the survival rates of patients [59]. An increase in T-BET-positive infiltrating lymphocytes in esophageal carcinoma (ESCA) and GC tissues remarkably correlates with the postoperative prognosis of patients [59]. T-BET expression in the intertumoral lymphoid tissue suggests a good prognosis with significantly improved recurrence-free survival in patients with human epidermal growth factor receptor 2 (HER2)-overexpressing breast cancer who underwent neoadjuvant trastuzumab-paclitaxel therapy [60, 61]. Patients with skin cutaneous melanoma (SKCM) and increased T-BET expression have a good prognosis. T-BET may be a promising prognostic biomarker for SKCM [62]. High TBX4 expression levels had significantly longer survival times than those showing low expression levels in patients with pancreatic ductal adenocarcinoma (PDAC) [63].

In addition to the association of T-BOX with the survival rate and prognosis of cancer, T-BOX is also related to the histological grade and cancer stage. For example, TBX2 expression was positively correlated with the TNM stage of ESCA [64]. Kandimalla et al. [65] reported that TBX2 levels are specific prognostic markers for pTa staging in bladder cancer. The level of TBX3 increases in patients with HCC with an increase in the histological grade of HCC [66]. These findings suggest the potential use of T-BOX as a marker for clinical tumor staging and grading.

## Cofactors and epigenetic modifications of the T-BOX family

The role played by the T-BOX family in development and disease is also associated with binding cofactors and epigenetic reprogramming. Given the difficulty of directly targeting transcription factors, a comprehensive understanding of the molecular mechanisms of intracellular interactions of the T-BOX family could facilitate the development of targeted drugs against cofactors and epigenetic abnormalities.

### T-BOX factors binding with molecules into complexes creates synergies

**Fig. 3:**
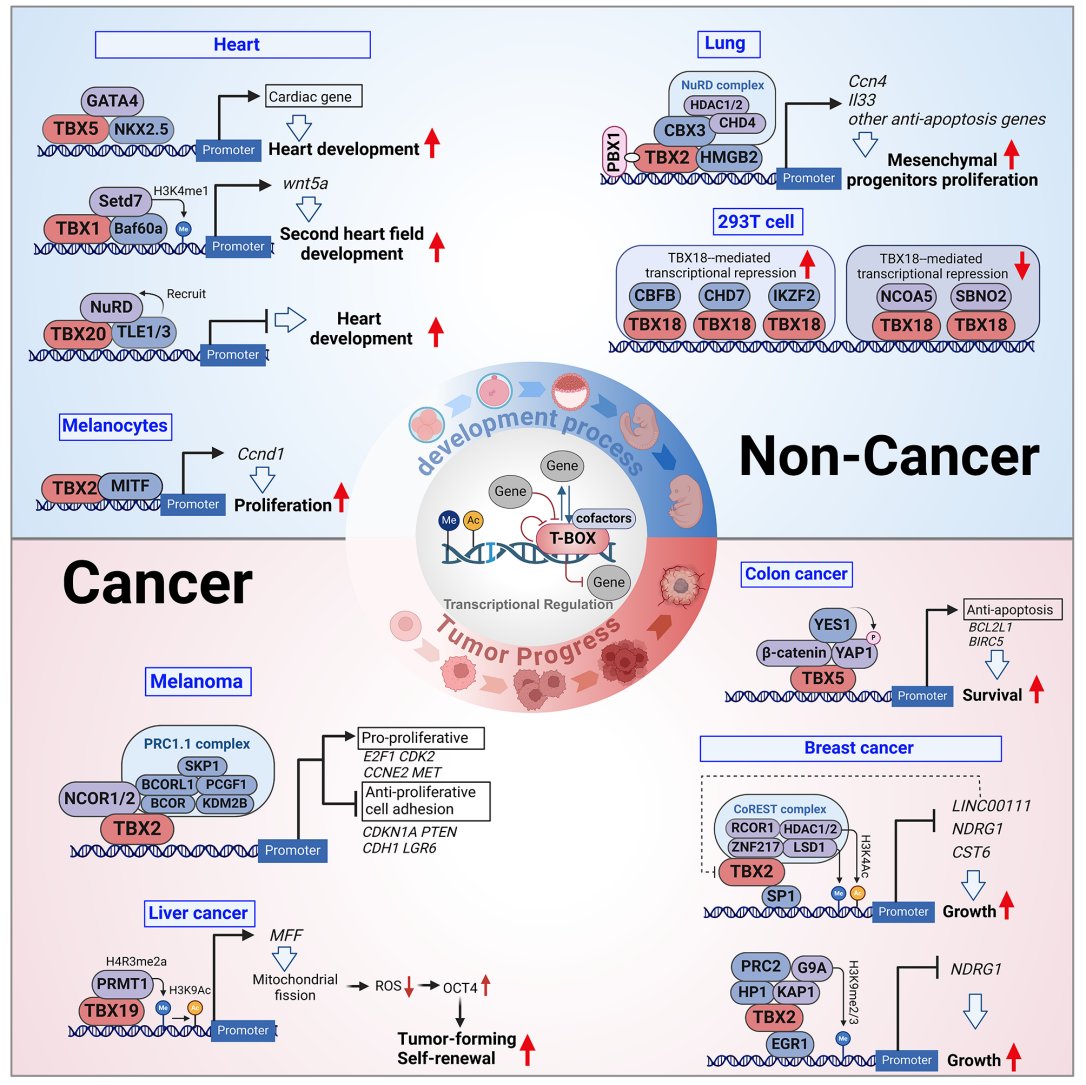
Binding of T-BOX transcription factors binding with other molecules to exert synergistic effects. T-BOX molecules recruit several molecules to form synergistic complexes during physiological and cancer progression. The T-BOX complexes in physiological development: during heart development, TBX5 binds to GATA4 and NKX2.5 to form complexes, TBX1 binds to SETD7 and BAF60a to synergistically promote *WNT5A* expression, and TLE1/3 recruits NuRD binding to TBX20 to form a complex. TBX2 binds to Microphthalmia-associated transcription factor (MITF) to upregulate Cyclin D1 (CCND1) expression and promote melanocyte proliferation. In lung development, TBX2 binds to the NuRD complex [including HDAC1/2 and DNA-binding protein 4 (CHD4)], Chromobox3 (CBX3), and High Mobility Group Box 2 (HMGB2) to synergistically promote the expression of anti-apoptotic genes such as Cellular Communication Network Factor 4 (*CCN4*) and Interleukin 33 (*IL33*), thereby promoting the proliferation of mesenchymal progenitors. In addition, in 293T cells, TBX18 binds to different molecules to enhance transcriptional promotion [Core-Binding Factor Subunit Beta (CBFB), Chromodomain Helicase DNA Binding Protein 7 (CHD7), and IKAROS Family Zinc Finger 2 (IKZF2)] or alleviate transcriptional repression [Nuclear Receptor Coactivator 5 (NCOA5) and Strawberry Notch Homolog 2 (SBNO2)]. T-BOX complexes in tumors: in breast cancer cells, TBX2 binds to CoREST protein complexes [including LSD1, ZNF217, histone deacetylase1/2 (HDAC1/2), and RCOR1] or G9A, PRC2, and HP1/KAP1, which synergistically suppress oncogenes (*CST6* and *NDGR1*) and promote tumor growth through epigenetic histone mediation. TBX2 binds to PRC1.1 complexes (including SKP1, BCORL1, PCGF1, BCOR, and KDM2B) and NCOR1/2 and exerts proliferative effects on melanoma cells. YAP1 and TBX5 form a complex with β-catenin and activate BCL2 Like 1 (*BCL2L1*) and Baculoviral IAP Repeat Containing 5 (*BIRC5*), thereby enhancing the anti-apoptotic effect of tumor cells. In hepatocellular carcinoma cells, TBX19 forms a transcriptional complex with PRMT1, eliciting epigenetic histone H4R3me2a/H3K9ac-mediated transactivation of Mitochondrial Fission Factor (*MFF*), which reduces ROS production and prevents ROS-mediated degradation of the pluripotent transcription factor OCT4, leading to enhanced tumor formation and cellular self-renewal

Synergy refers to the interaction and coordination between different proteins, working together to achieve a common function. In the case of the complex formed by the interaction of transcription factors with other proteins, synergy can enhance transcriptional activity and increase gene specificity, ensuring the normal functioning of cells and tissues during physiological and developmental processes. Several important T-BOX transcription factors and molecular synergy lead to transcriptional co-activation or inhibition (Fig. 3).

TBX1, TBX2, TBX5, TBX18, and TBX20 bind with several molecules to form complexes that synergistically regulate development. TBX2 promotes melanocyte proliferation by forming a complex with Microphthalmia-associated transcription factor (MITF) to upregulate Cyclin D1 (CCND1) expression [67]. During lung development, TBX2 binds to the nucleosome remodeling and deacetylase (NuRD) complex [including histone deacetylase1/2 (HDAC1/2) and DNA-binding protein 4 (CHD4)], Chromobox3 (CBX3), and High Mobility Group Box 2 (HMGB2). It synergistically upregulates anti-apoptotic genes expression such as Cellular Communication Network Factor 4 (*CCN4*) and Interleukin 33 (*IL33*), promoting the proliferation of mesenchymal progenitor cells [68]. Many cofactors are involved in the important role of T-BOX factors in heart development. TBX1 binding to the SET domain containing lysine methyltransferase 7 (SETD7) and BRG1-associated factor 60a (BAF60a) synergistically promotes *WNT5A* expression to improve second heart field development. TBX5 binds to GATA binding protein 4 (GATA4) and NK2 homeobox 5 (NKX2.5) to genes related to heart development. TBX5 combined with myocyte enhancer factor 2 C (MEF2C) to transcriptionally activate Myosin Heavy Chain 6 (*MYH6*). TLE1/3 recruits the NuRD complex to bind to TBX20, which form a transcriptional inhibition complex [69–72]. TBX2 binding with NKX2.5 synergistically inhibits atrial natriuretic factor (ANF) expression, thereby suppressing cardiac development [73, 74]. The close association of TBX18 with the development of the ureter has prompted scientists to study its function in depth. In 293T cells, different molecules bind to TBX18 to enhance transcriptional repression [Core-Binding Factor Subunit Beta (CBFB), Chromodomain Helicase DNA Binding Protein 7 (CHD7), and IKAROS Family Zinc Finger 2 (IKZF2)] or to alleviate transcriptional repression [Nuclear Receptor Coactivator 5 (NCOA5) and Strawberry Notch Homolog 2 (SBNO2)] [75], suggesting that the expression of cofactors may be associated with TBX18-related ureter development and urological diseases. The same T-BOX family member may have different or even opposite effects if the factors binding or interacting with are different. This phenomenon reflects the complex and vital role of the T-BOX family in physiological development. Therefore, even slight dysregulation of the T-BOX family can potentially lead to severe diseases, including cancer.

T-BOX molecules act synergistically in combination with other molecules during tumor progression. TBX2 complex plays a synergistic role in tumor growth. For example, in breast cancer, two TBX2 complexes have been identified: the TBX2-CoREST complex targets N-Myc Downstream Regulated 1 (*NDRG1*) by recruiting TBX2 to the *NDRG1* promoter via Sp1 transcription factor (SP1). The TBX2-CoREST complex catalyzes the closure of chromatin around the target promoter via LSD1 demethylation and HDAC1/2 deacetylation, inhibiting tumor suppressor targets (Long Intergenic Non-Protein Coding RNA 111 [*LINC00111*], *NDRG1*, and Cystatin E/M [*CST6*]); The TBX2- KRAB associated protein 1(KAP1)- Heterochromatin Protein 1 (HP1) complex drives G9A-dependent H3K9 methylation and interacts with early growth response factor 1 (EGR1) to inhibit *NDRG1* [76–78]. In melanoma, TBX2 binding with the PRC1.1 complex and nuclear receptor corepressor 1/2 (NCOR1/2) exerts anti-senescence and proliferation-promoting effects on tumor cells [76]. TBX5 and YES-associated protein 1 (YAP1) form a complex with β-catenin, which localizes and activates BCL2 Like 1 (*BCL2L1*) and Baculoviral IAP Repeat Containing 5 (*BIRC5*), thereby enhancing the anti-apoptotic effect on tumor cells [79]. In HCC, TBX19 forms a transcriptional complex with protein arginine methyltransferase 1 (PRMT1), eliciting epigenetic histone H4R3me2a/ H3K9ac-mediated transactivation of Mitochondrial Fission Factor (*MFF*), which prevents reactive oxygen species (ROS)-mediated degradation of the pluripotent transcription factor OCT4, leading to enhanced tumor formation and cellular self-renewal [80]. Given that drug therapy targeting transcription factors remains a huge challenge, targeting these molecules and thereby inhibiting the formation of the T-BOX transcription complex is a promising manner. This targeted therapy reduces the synergistic transcriptional effects and thus inhibits cancer progression to some extent. It provides new ideas for the targeted therapy of T-BOX-positive cancers.

### DNA methylation and histone modifications in T-BOX family

In recent years, a boom has been observed in research related to various epigenetic regulatory models, including DNA methylation and histone modifications. Epigenetic effects are significant in randomly occurring tumor development and cancer progression. The T-BOX family has been found to involve in epigenetic modifications during developmental and tumor processes. Notably, the same T-BOX has different epigenetic modifications in various microenvironments that inhibit or promote its transcriptional activity, thereby activating different target genes and leading to different outcomes. For example, TBX5 is involved in different epigenetic modifications. In early cardiac development, TBX5 histones deacetylation mediated by the class II histone deacetylases HDAC4 and HDAC5 increases the gravitational force between DNA and histones, making the relaxed nucleosomes very tight and inhibiting the expression of *TBX5* gene, limiting premature differentiation of cardiac cells and allowing normal heart development [81, 82]. However, in colon cancer, hypermethylation of the CpG island of the *TBX5* promoter results in the inability of the promoter region to bind transcription-associated proteins, inhibiting *TBX5* transcriptional activity leads to the development of malignant tumors [57].

Transcriptional downregulation in early colorectal cancer tissues due to hypermethylation of the *TBX20* promoter is linked to poor prognosis in colorectal cancer patients [83]. *TBR1* DNA methylation frequently occurs in urologic cancer cell lines, particularly in three types of solid tumors in the urinary tract, renal, uroepithelial, and prostate cancer, and is involved with poor prognosis of urologic tumors [84]. *TBX1* promoter methylation in thyroid cancer results in the downregulation of TBX1 expression, promoting cancer progression. Interestingly, *TBX1* methylation is significantly correlated with gender, explaining to some extent the higher incidence of thyroid cancer in women [85]. Compared to normal alveolar epithelial cells, NSCLC cell lines exhibit DNA methylation and suppress mRNA expression in the *TBX2* subfamily members. High methylation in the *TBX2* subfamily occurs early in the development of NSCLC, making it a potential biomarker that can be utilized [86].

HDAC3 deacetylates TBX5 and represses TBX5 transcriptional activity to maintain the pluripotent state of cardiac progenitor cells. HDAC3 and E1A Binding Protein P300 (EP300) regulate the acetylation of TBX5 and identify Lys157 and Lys159 as conserved acetylation sites for TBX5 [81]. Recruitment of HDAC1 and HDAC3 by TBXT is critical in the formation of the primitive streak as well as gastrulation [87].

### Post-translation modifications in T-BOX family

The phosphorylation of TBX3 at the serine-proline motif at amino acid 190 (SP190), S692 and S720 sites has been revealed [88–90]. TBX3 is phosphorylated by p38 MAPK kinase at S692 in mouse and AKT3 at S720, respectively, and can inhibit transcription of the downstream target E-cadherin. During melanoma development, the phosphorylation at S720 via AKT3 resulted in increased TBX3 protein stability and nuclear localization, which represses the E-cadherin expression leading to cell migration and invasion [89]. While phosphorylation of TBX3 at SP190 was found to inhibit the downstream p21 promoter in chondrosarcoma, the kinase responsible for phosphorylation at SP190 has not been identified. However, it has been hypothesized that extracellular signal-regulated kinase (ERK) is more likely [90]. The E3 ubiquitin ligase PDZ Domain Containing Ring Finger 3 (PDZRN3) mediates the ubiquitination and degradation of TBX20 protein in early colorectal cancer tissues. Inhibition of PDZRN3 restores the down-regulated TBX20 expression [83], which provides an idea for finding targets for cancer therapy.

## Multiple roles of T-BOX factors in cancers

According to numerous studies, T-BOX factors play vital roles in EMT, stemness, apoptosis, tumor proliferation, and drug resistance during tumor progression, which have significant implications for cancer diagnosis and treatment. Moreover, the same T-BOX member may function as tumor-promoting or tumor-suppressing molecule depending on the tumor microenvironment. For example, TBX1 plays a tumor-promoting role in basal cell carcinoma and TBX3 plays a tumor-promoting role in basal cell carcinoma in HCC, melanocytoma, and bladder cancer, whereas TBX1 and TBX3 exert a tumor-suppressing effect on thyroid cancer and fibrosarcoma, respectively [85, 91–96].

### EMT

**Fig. 4:**
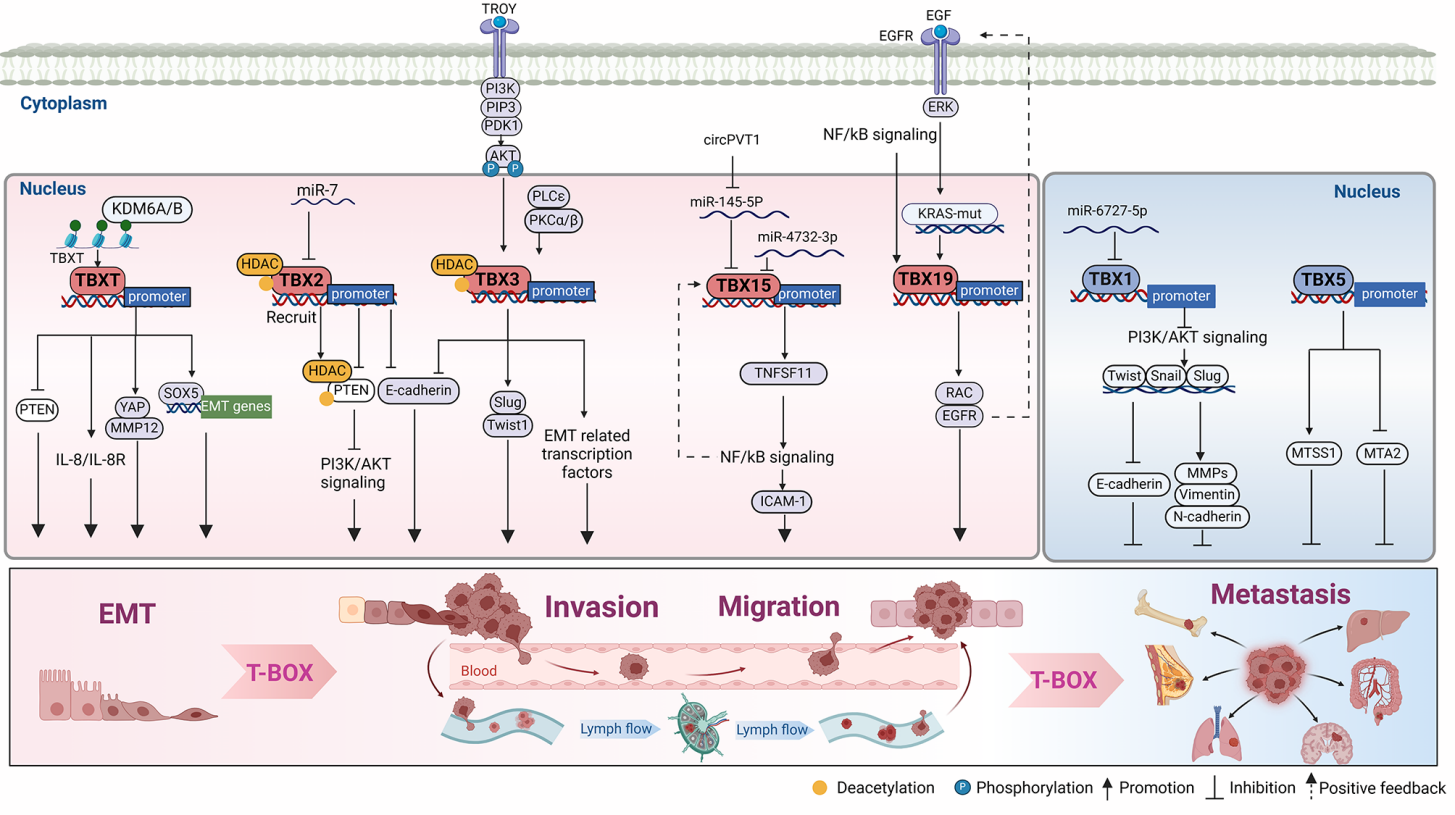
T-BOX transcription factors act as critical nodes in tumor metastasis. T-BOX members serve as master tumor activators or suppressors to transcriptionally regulate key downstream targets or signaling pathways in tumor metastasis. The dotted line indicates positive feedback. T-BOX primarily influences tumor progression by promoting or inhibiting epithelial–mesenchymal transition (EMT). Activator: TBXT leads to pro-EMT gene expression (Snail, vimentin, N-cadherin, and fibronectin) via transcriptional activation of *YAP1*, *MMP12*, *SOX5*, and *IL-8/IL-8R*. TBX2 directly inhibits PTEN, induces EMT-related gene expression, and reduces E-cadherin expression. TBX3 expression is regulated by PKCα/β and the TROY/PI3K/AKT/TBX3 axis. TBX3 promotes *SLUG* and *TWIST1* expression, represses *PTEN*, and upregulates EMT related gene expression. TBX15 promote tumor metastasis through the NF-kB pathway and form a positive feedback loop. The epidermal growth factor (EGF)/ EGF receptor (EGFR) signaling pathway upregulates TBX19 expression via the ERK/NF-kb axis as well as KRAS mutations, and TBX19 upregulates EGFR and Rac expression to promote cancer metastasis, forming a positive feedback loop. Suppressor: *miR6727-5p* inhibits *TBX1* expression, and TBX1 suppresses cancer cell migration and invasion by downregulating the expression of EMT-related genes (*TWIST*, *Snail*, and *SLUG*) and *MMPs* (*MMP12*, *MMP9*, and *MMP14*). TBX5 upregulates MTSS I-BAR Domain Containing 1 (*MTSS1*) expression and inhibits Metastasis Associated 1 Family Member 2 (*MTA2*) expression, exerting its inhibitory effect on cancer cell metastasis

EMT can promote carcinogenesis through various mechanisms, including the conferment of migratory and invasive properties to cells. The mesenchymal status is associated with the capacity of cells to invade and migrate to distant organs [97]. The cancer-promoting effects of T-BOX factors are associated with the EMT process. T-BOX factors affect the EMT process directly or indirectly via the regulation of their downstream molecules (Fig. 4).

TBXT is a critical regulatory EMT molecule involved in tumor metastasis and invasion. TBXT promotes bone metastasis in breast cancer by transcriptionally activating oncogene SRY-Box transcription factor 5 (*SOX5*) [40]. In patients with breast cancer, SOX5 binds to the promoter of *TWIST1* (a significant regulator of embryonic morphogenesis), thereby promoting its expression and facilitating EMT for metastasis [98]. TBXT inhibits the phosphatase and tensin homolog PTEN (a factor that negative regulates the expression of Snail, a key mediator of EMT) [99, 100], promoting EMT progression. In patients with SCC, overexpression of TBXT inhibits E-cadherin (epithelial phenotype cell biomarker) expression, which induces tumor migration and invasion [38]. Furthermore, overexpression of TBXT induces EMT in a variety of human cancer cells (breast, pancreatic, and lung) and is associated with enhanced secretion of various cytokines, chemokines, and angiogenic factors, especially with induction of the IL-8/IL-8R axis in tumor cells. IL-8 signaling plays an important role in acquiring and maintaining mesenchymal and invasive characteristics of TBXT overexpressing tumor cells, and IL-8 secreted by tumor cells during EMT can induce phenotypic transformation of neighboring epithelial tumor cells and acquire metastatic potential [101]. TBX1 downregulates the expression of matrix metalloproteinases (MMPs), exerting a EMT-inhibitory effect in thyroid cancer cells [85].

TBX2 expression is also significantly associated with EMT. Specifically, TBX2 leading to EMT is modulated by the downregulation of *miR-7* in GBM, resulting in increased vimentin expression and decreased E-cadherin expression, causing an increase in the number of invading cells and lung metastases [47]. Ectopic expression of TBX2 in mammary epithelial cells induces changes in molecular, behavioral and morphological characteristics of EMT, including epithelial adhesion, loss of expression of polarity-related genes (β-catenin, E-cadherin, and Zona occludens 1 [ZO1]), abnormal increases in mesenchymal marker levels (N-cadherin and vimentin), and increased cell motility and cancer invasion [48].

Overexpression of TBX3 increases cell survival, growth, and invasiveness as it regulates genes associated with EMT, including *SLUG*, *TWIST1* and E-cadherin [102, 103]. For instance, *TROY* [liver progenitor cell-specific gene tumor necrosis factor (TNF) receptor superfamily member 19] causes p85α degradation, which consequently activates the PI3K/AKT/TBX3 axis and leads to induction of EMT in HCC cells [94]. TBX3 directly promotes the expression of Inhibitor of DNA binding 1 (ID1), a key regulator of tumor progression, and inhibits E-cadherin expression via the TBX3-ID1-MITF-axis, promoting migration and invasiveness of melanoma cells [91, 92, 104].

### Invasion, migration, and metastasis

In addition to the EMT process, the T-BOX family also promotes the invasion and metastasis of tumors through other means (Fig. 4) TBXT significantly upregulates MMP12 expression [41] (a key protein for invasive and migration potential in various malignancies [55]) helping tumor cells in acquiring and maintaining mesenchymal and invasive characteristics.

The inhibition of E-cadherin by TBX3 is also regulated by protein kinase C α/β (PKCα/β), which is engaged in the Phospholipase C-ε (PLCε)-mediated migration and invasion of bladder tumor cells [95]. In addition, TBX3 is activated by AKT1 in fibrosarcoma cells. TBX3 directly binds to and activates collagen type I alpha 2 chain (*COL1A2*), mediating the inhibitory effect of TBX3 on fibrosarcoma cell migration [96].

Recently, our research has revealed that TBX19 overexpression promotes HCC metastasis via the upregulation of EGFR and Rac1 expression. The EGF/ EGFR signaling pathway upregulates the expression of TBX19 via the ERK/ nuclear factor (NF)-kb axis [5]. Thus, the EGF/TBX19/EGFR positive feedback pathway is vital for HCC progress. EGFR is associated with cell survival, differentiation, and proliferation as it binds to its ligand, EGF, whose structural activation promotes various human cancers metastasizing [105]. In addition, TBX19, activated by *KRAS* mutations, is upregulated in colon and rectal cancers and is associated with lymph node metastasis [106–108].

TBX15 has a pro-carcinogenic effect. TBX15 upregulates the expression of TNFSF11. TNFSF11 activates the NF-κB signaling pathway and upregulates the expression of ICAM-1 and MEK/ERK phosphorylation, thereby promoting lung cancer migration [109]. miR-4732-3p negatively regulates TBX15 expression by binding to the 3’- untranslated regions (UTR) of TBX15 mRNA [109]. *TBX15* mRNA expression is higher in ccRCC tissues than in normal tissues, and overexpression of TBX15 leads to the migration and the invasion of ccRCC cells [6]. Zheng et al. found that TBX15 expression is stimulated by CircRNA plasmacytoma variant translocation 1 (circPVT1), which suppresses the inhibitory effect of *miR-145-5p* on *TBX15* by directly binding to it [6]. Methylation of the *TBR1* gene is associated with tumorigenesis and cell migration. Serth et al. [84] observed increased DNA methylation in a genomic region corresponding to the 3′UTR of the *TBR1* mRNA in both non-metastatic and metastatic tumor tissues. It is believed that cells carrying the methylation mark are progressively enriched because of the initiation, progression, and metastasis of tumors [110]. This observation is consistent with the finding that *TBR1* methylation promotes cancer cell growth and metastasis [84]. *TBX22*, a tumor suppressor gene regulating papillary thyroid carcinoma (PTC), suppresses tumor proliferation and migration [111]. Dong et al. [111] observed mutations in B-Raf proto-oncogene (*BRAF*^*V600E*^) and telomerase reverse transcriptase (*TERT*)(specific molecules that can be used to predict the risk of extrathyroid invasion and lymph node metastasis) in PTC, resulting in significant downregulation of TBX22. Functionally, overexpression of TBX22 inhibits the proliferation and migration of PTC cells. However, the role of TBX22 in tumors has not been comprehensively described. TBX22 deserves further exploration as a potential target for governance based on its tumor suppressor properties TBX5 has an inhibiting effect on cancer cells by upregulating MTSS I-BAR Domain Containing 1 (*MTSS1*) or suppressing the expression of the pro-metastatic gene Metastasis Associated 1 Family Member 2 (*MTA2*) [57].

### Tumor stemness

**Fig. 5:**
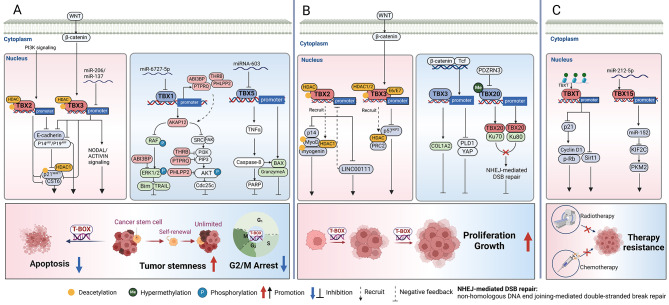
T-BOX transcription factors act as critical nodes in tumor progress. T-BOX members serve as master tumor activators or suppressors as they transcriptionally regulate key downstream targets or signaling pathways, thereby controlling the progression of cancer, including stemness, apoptosis, proliferation and growth, and therapy resistance. **A** Activator: *TBX2* expression was regulated by NRAGE and PI3K signaling. TBX2 and TBX3 enable cells to bypass senescence via the p14^ARF^/p19^ARF^-p53-p21 signaling pathway and inhibit apoptosis. *TBX3* expression was inhibited by *miR-206/miR-137* and WNT/β-catenin signaling pathway. TBX3 induces the CSC phenotype by regulating NODAL/ACTIVIN signaling. Suppressor: TBX1 inhibits phosphorylation in AKT and ERK pathways and promotes the expression of the pro-apoptotic genes *BIM*, *TRAIL*, and *CDC25C* by upregulating *AKAP12*, *THRB*, *ABI3BP*, *PTPRQ*, and *PHLPP2* expression, thereby inducing G2/M cell cycle arrest and apoptosis. TBX5 induces apoptosis by targeting extrinsic pathways (TNFα /TNFRSF10B/TNFRSF1A/TNFRSF25-Caspase8), thereby enhancing the expression levels of PARP and apoptotic gene *BAX* and the Granzyme A signaling cascade; **B** Activator: TBX2 recruits HDAC and interacts with MyoD and myogenin to promote tumor progression. TBX3 recruits PRC2 and HDAC chromatin modification complexes to suppress p57^KIP2^ expression. Suppressor: TBX3 activates *COL1A2* or inhibits the PLD1/YAP axis to inhibit tumor progression. TBX20 inhibits tumor growth inhibition by binding to the intermediate structural domains of Ku70 and Ku80, which inhibit NHEJ-mediated DNA repair. PDZRN3 mediates degradation of TBX20; **C** TBXT increases tumor resistance by inhibiting SIRT1 expression and the p21/Cyclin D1/pRb pathway. TBX15 expression is inhibited by *miR-212-5p*, which is regulated by the NF-kb axis, and this process results in an increase in tumor resistance via the *miR-152*/ Kinesin Family Member 2 C (KIF2C) /PKM2 pathway

Tumor stemness refers to the presence of a specific type of stem cell within a tumor, known as cancer stem cells (CSCs). CSCs are a unique subpopulation of tumor cells with an unlimited potential for self-renewal. CSCs promote tumorigenesis, facilitate metastasis, and enhance resistance to cancer therapy [112]. Since T-BOX molecules exhibit an important role in cell proliferation, they are destined to participate in the stemness of tumors. (Fig. 5A). For example, TBX3 is important for stem cell self-renewal and has been found to play an important role in cancer stemness. TBX3 can be used as a stem cell marker for many cancer types [30].

*TBX3* has been identified as a target of tumor suppressor *miR-206*. In breast cancer cells, *miR-206* inhibits the expression of the *TBX3* gene by directly binding to it, thereby suppresses the cancer stem cell population [113]. Ectopic TBX3 expression enhances the formation of breast cancer tumorspheres, which are generated by the proliferation of individual CSCs [114]. TBX3-overexpressing cancer cells in ERα breast cancer cell lines promote CSCs expansion through paracrine FGF signaling. Mechanistically, estrogen binds to the estrogen receptor and induces FGF9 secretion and TBX3 expression in non-CSCs compartments. TBX3 expression leads to further expression of Wnts and FGF, which ultimately results in the expansion of CSCs in terms of area and number. In contrast, the knockdown of *TBX3* significantly reduces the number of CSCs in various breast tumor cell lines and diminishes their ability to form tumor spheres [114]. In pancreatic cancer, TBX3 activates NODAL/ACTIVIN signaling to promote CSCs self-renewal [115]. NODAL and ACTIVIN are crucial regulators of embryonic stem cell fate. Studies have shown that NODAL/ACTIVIN signaling is essential for cancer continuation and tumorigenic potential [116]. BRAF^V600E^-activated MAPK signaling maintains high proliferative capacity by transcriptionally upregulating ubiquitin-specific protease (USP)15 and protecting TBX3 from degradation [117]. Due to the dysplasia in TBX3 deficiency and the tumorigenic risk of high TBX3 levels, strict control of its expression levels is required. Key mediators of embryogenesis are frequently reactivated during cell transformation and tumorigenesis. For example, three pluripotent transcription factors, OCT4, NANOG, and SOX2, are aberrantly expressed in different types of tumors and trigger molecular reconfigurations of epigenomic landscapes similar to those of embryonic stem cells, suggesting a common mechanistic link between embryogenesis and tumorigenesis. Key morphogenetic and oncogenic signaling pathways such as PI3K, TGF-β, FGF, WNT/β-catenin, and BRAF/MAPK are involved in the regulation of T-BOX expression. T-BOX, as a family of key developmentally relevant transcription factors, is essential for the maintenance of embryonic stem cell identity, cell lineage differentiation, and organogenesis. T-BOX molecular expression is finely regulated, and aberrant expression levels may lead to aberrant regulation of cell fate and proliferation, which in turn can lead to a variety of malignancies. Within precisely regulated cells, numerous factors may interfere with T-BOX expression leading to an immediate transition from normal development to carcinogenesis. How the T-BOX family is currently reprogrammed from development to the cancer spectrum still needs to be explored in depth.

### Anti-apoptosis

Apoptosis is a negative growth regulatory mechanism in cancer cells, and its inhibition is one of the causes of cancer development. The mechanisms of apoptosis are complex and involve many pathways that play an important role in the malignant transformation of cells and tumor progression [118], including intrinsic (mitochondrial), extrinsic (death receptor), and intrinsic endoplasmic reticulum pathways [118], among which, PI3K/AKT and MAPK/ERK pathways are closely related to T-BOX factors [85]. (Fig. 5A).

TBX1 is thought to suppress cancer cell growth in mouse skin tumors [119]. Furthermore, overexpression of TBX1 significantly suppressed cell viability and tumorigenic potential in thyroid cancer cells of nude mice. It induced apoptosis by regulating the cell cycle and apoptosis-related genes, significantly reducing the migratory and invasive potential of thyroid cancer cells [85]. Mechanistically, TBX1 induces G2/M cell cycle arrest and apoptosis by upregulating genes inhibiting the PI3K/AKT and the MAPK/ERK pathways, such as A-Kinase anchoring protein 12 (*AKAP12*), thyroid hormone receptor beta (*THRB*), ABI family member 3 binding protein (*ABI3BP*), protein tyrosine phosphatase receptor type Q (*PTPRQ*), PH domain and leucine-rich repeat protein phosphatase 2 (*PHLPP2*). These factors inhibit AKT phosphorylation at Ser473 and ERK phosphorylation [85].

*TBX5* is an oncogene and is associated with cancer cell apoptosis. YAP1 and TBX5 form a complex with β-catenin, which localizes to the promoter of anti-apoptotic genes, enhancing their anti-apoptotic effects in tumor cells [79]. In addition, TBX5 may exert its inhibitory effects on cancer cells by targeting the extrinsic pathway, apoptotic gene *BAX*, and Granzyme A signaling cascade to induce apoptosis [57]. Furthermore, TBX2 and TBX3 represses the tumor suppressor genes p14^ARF^ (human)/p19^ARF^ (mouse) and p21 during tumorigenesis. Inhibition of the p14^ARF^ (human)/p19^ARF^ (mouse)-p53-p21 signaling pathway allows cells to bypass senescence and inhibit apoptosis [104, 120]. T-BET is a tumor suppressor significantly downregulated in colorectal cancer (CRC) tissues [121]. T-BET directly binds to the Rho GTPase Activating Protein 29 (*ARHGAP29*) promoter, leading to the inhibition of the Glycogen synthase kinase-3 (GSK3) signaling pathway. T-BET promotes apoptosis and suppresses cell proliferation, ultimately inhibiting the progression of colorectal tumors [121].

### Proliferation and growth

High rates of proliferation and rapid growth are intrinsic nature of aggressive tumors. Understanding the mechanisms underlying these processes in tumors can provide a basis for targeting these pathways. Therefore, we have summarized the effects of T-BOX transcription factors on the growth and proliferation of cancer cells (Fig. 5B).

The transforming growth factor-β (TGF-β)/SOX6/SOX9/TBXT pathway appears to play a vital role in the occurrence, survival, and proliferation of chordomas [122]. This network exhibits high interdependence, with TBXT playing a critical role. The pro-cancer mechanism of TBX2 primarily occurs through the induction of cells to bypass senescence. Endogenous TBX2 is critical for maintaining melanoma cell proliferation and inhibiting senescence [123]. Mechanistically, TBX2 in cell proliferation is partly mediated via the regulation of *p21* and cyclin E (accumulating at the G1/S phase transition to accelerate cell proliferation) expression in GC cells [55]. TBX2 promotes *E2F1* expression in melanoma by directly binding to it, a key anti-aging cell cycle regulator. PI3K signaling can increase the activity of TBX2 that promote bypass of senescence; however, the specific mechanism requires further analysis [76]. *TBX2* is an oncogene in rhabdomyosarcoma. TBX2 recruits HDAC1 and interacts with myogenic regulators MyoD and myogenin, promoting rhabdomyosarcoma cell proliferation by inhibiting *p21* [124]. In addition, TBX2 is a key oncogenic molecule in breast cancer. TBX2 forms complexes with critical molecules synergistically exerting cancer-promoting effects [77, 78].

TBX3 promotes cell proliferation and tumorigenesis in PTC and hypopharyngeal carcinoma by promoting the G1/S transition in the cell cycle. TBX3 recruits PRC2 and HDAC1/2 chromatin modification complexes to bind directly to the Cyclin Dependent Kinase Inhibitor 1 C (*CDKN1C*) gene promoter region (encoding the critical cyclin-dependent kinase inhibitor p57^KIP2^) and represses its transcription [125, 126]. *TBX3* generally is considered an oncogene in HCC [102]; however, a recent study found that TBX3 plays a tumor-inhibitory role in HCC [127]. *CTNNB1* (encoding β-catenin) mutation induces TBX3 expression, which in turn inhibits phospholipase D1 (PLD1) and YAP/TAZ pathway activation, suppressing cancer proliferation and growth. TBX3 is regulated by AKT1 and plays a cancer-suppressive role in fibrosarcoma; TBX3 directly binds to the promoter of *COL1A2*, inhibiting substrate-dependent and non-substrate-independent cell proliferation, migration, and in vivo tumorigenic capacity [96]. Furthermore, TBX3 is expressed in human papilloma virus (HPV)-negative cervical cancer cells and inhibits tumor proliferation and migration. However, in HPV-positive cervical cancer, TBX3 collaborates with E6/E7 oncogenic proteins to promote cancer cell proliferation, colony formation, and migration [128]. In conclusion, TBX3 may act as a tumor promoter or suppressor, depending on the cellular context [129]. Therefore, it is important to understand the molecular mechanisms that enable the switch between these effects.

The inhibitory effect of TBX20 on tumor growth occurs through the suppression of non-homologous DNA end-joining (NHEJ)-mediated DNA repair in CRC cells. TBX20 inhibits the recruitment of Ku70 and Ku80 to the chromatin of CRC cells, inhibiting NHEJ-mediated double-strand break repair, thereby suppressing cell proliferation and tumor growth. However, low expression of PDZRN3 in tumors leads to increased TBX20 degradation [130].

TBX1 is upregulated in PCa tissues [54]. Silencing of *TBX1* inhibited PCa cell proliferation and colony formation by leading cells arrest in the G0/G1 phase. In addition, TBX1 silencing reduces enrichment of the rRNA genes H3K4me1 and Upstream Binding Factor (UBF) (key transcriptional regulators of rRNA synthesis) and inhibits PCa progression, suggesting that TBX1 may affects tumor progression in this epigenetic manner [54]. However, whether TBX1 promotes tumor progression by increasing the enrichment of H3K4me1 as well as UBF on rRNA genes is still unclear, and further studies are needed to elucidate these specific mechanisms. The involvement of TBX5 in cell proliferation has been observed. TBX5 is regulated by the YAP1/β-catenin pathway and is required for the proliferation and survival of CRC cells [131]. However, TBX5 functions as a tumor suppressor in colon cancer cells by inhibiting cell proliferation and inducing apoptosis [57]. Mechanistically, TBX5 upregulates the expression of the key cell cycle inhibitory gene Cyclin Dependent Kinase Inhibitor 2 A (*CDKN2A*) and inhibits oncogenic Synuclein Gamma (*SNCG*) expression [57].

### Therapy resistance

There are many key factors of tumor resistance, including tumor growth kinetics, tumor heterogeneity, tumor burden, physical barriers, immune system and tumor microenvironment, and undruggable drivers of cancer [132]. Resistance to molecularly targeted therapies and chemotherapy is a significant challenge in current cancer research. Comprehensive understanding of the mechanisms underlying tumor cell drug resistance could provide additional help in developing and improving new therapeutic modalities. Several T-BOX transcription factors have been found to influence tumor resistance by regulating oncogenes; here, we summarize the different mechanisms of T-BOX family affecting tumor cell resistance to chemotherapy drugs (Fig. 5C).

Overexpression of TBXT in human lung cancer cell lines is positively associated with resistance to EGFR kinase inhibitors [7]. In a previous study, the knockdown of *TBXT* significantly decreased the drug resistance of A549 cells to cisplatin (CDDP) [8]. In addition, TBXT mediates tamoxifen resistance by silencing the expression of sirtuin-1 (*SIRT1*), an oncogene that has been observed to be overexpressed in a variety of cancers [133]. TBXT expression resulted in poor prognosis in breast cancer patients treated with tamoxifen [134, 135]. TBXT overexpression results in significant downregulation of *p21*, *CCND1* (encoding cyclin D1), and phosphorylated Rb (*p-Rb*), which increases lung cancer resistance to radiotherapy and chemotherapy [136]. Gemcitabine (GEM) is a pyrimidine antimetabolite that inhibits DNA synthesis in cancer cells by converting it to triphosphate. Recently, TBXT has been found to contain epitopes that stimulate CD4 + helper T lymphocytes (HTLs), which can exert a direct antitumor effect by inducing antigen-specific HTLs. Anti-PD-1 antibodies and GEM can enhance the reactivity of TBXT-reactive T cells in tumor. Therefore, TBXT peptide combination with GEM and immune checkpoint blockade may be promising immunotherapy for head and neck SCC [43].

TBX1 is downregulated in CC tissues targeted by *miR-6727-5p*, which inhibits CC progression, enhances chemosensitivity to cisplatin, and improves prognosis [137]. TBX2 is associated with cellular resistance. Silencing of *TBX2* in cisplatin-resistant breast tumor tissues disrupts the ATM-CHK2-p53 signaling pathway, increasing the drug sensitivity of breast tumor to cisplatin. It suggests that combining targeted therapy against TBX2 with chemotherapeutic agents such as cisplatin can enhance the effectiveness of current anti-cancer treatments [138]. TBX15 inhibits Kinesin Family Member 2 C (KIF2C) (a key microtubule regulator important for late chromosomal segregation, promoting solid tumor growth, metastasis, and chemotherapy resistance by modulating *p53* or inhibiting microtubule dynamics) expression via upregulation of *miR-152*. TBX15 blocks autophagy and glycolysis in doxorubicin (DOX)-resistant breast cancer cells, thereby regulating PKM2 ubiquitination. PKM2 is a glycolytic enzyme and significantly upregulated in many cancer tissues, promoting cell proliferation, migration, and metastasis through enhanced glycolysis. TBX15 reduces PKM2 stability to mediate doxorubicin resistance in breast cancer [9].

In summary, T-BOX molecules play a crucial role in developing various types of cancers. However, the same T-BOX molecule can have different effects in different cancers, possibly due to the different microenvironments of the cancers. For example, TBX3 promotes melanoma, bladder cancer, and liver cancer but inhibits fibrosarcoma. There are significant differences between cancer and sarcoma regarding cell origin and characteristics, resulting in the opposite effects of TBX3. However, more detailed and systematic studies still need to be completed to understand this intriguing phenomenon further. Moreover, TBX5 molecules also exhibit such duality, likely due to their interaction with different cofactors, thereby altering their functions. In conclusion, the tumor microenvironment is a complex and interconnected place. Uncovering the context-dependence of T-BOX family helps reveal the heterogeneity of the tumor microenvironment.

The impact of the T-BOX family on cancer extends throughout tumor progression, from tumorigenesis, to proliferation and metastasis, where the T-BOX family undergoes expression deregulation under the influence of various microenvironmental reprogramming, leading to transcriptional dysregulation. Throughout cancer progression, the T-BOX family has been found to be closely associated with key oncogenic pathways such as NF-kB, WNT/β-catenin, EGF\EGFR. The T-BOX family forms a complex regulatory network with key oncogenes, inflammatory factors, invasive metastasis-associated molecules, and pro-metastasis-associated molecules such as IL-8, MMP12, TNFSF11 and YAP. Tumor progression is a very complex process in which differential expression of the T-BOX family and epigenetic changes result in alterations in the tumor microenvironment need more explore. Restoration of normal T-BOX expression is the key to treating T-BOX disorder-associated cancers. It is very promising to utilize more modern and new technologies such as single-cell technology to develop targeted T-BOX molecule-associated drugs in different types of tumors. In addition, based on the fact that many studies have found that T-BOX factors is associated with resistance to chemotherapy or immune checkpoint inhibitors, future research directions are focused on combining targeted T-BOX drugs with other antitumor agents to reduce resistance to tumor therapy and thereby improve prognosis.

## T-BOX transcription factors in immunity: from physiology to tumor immunity

**Fig. 6:**
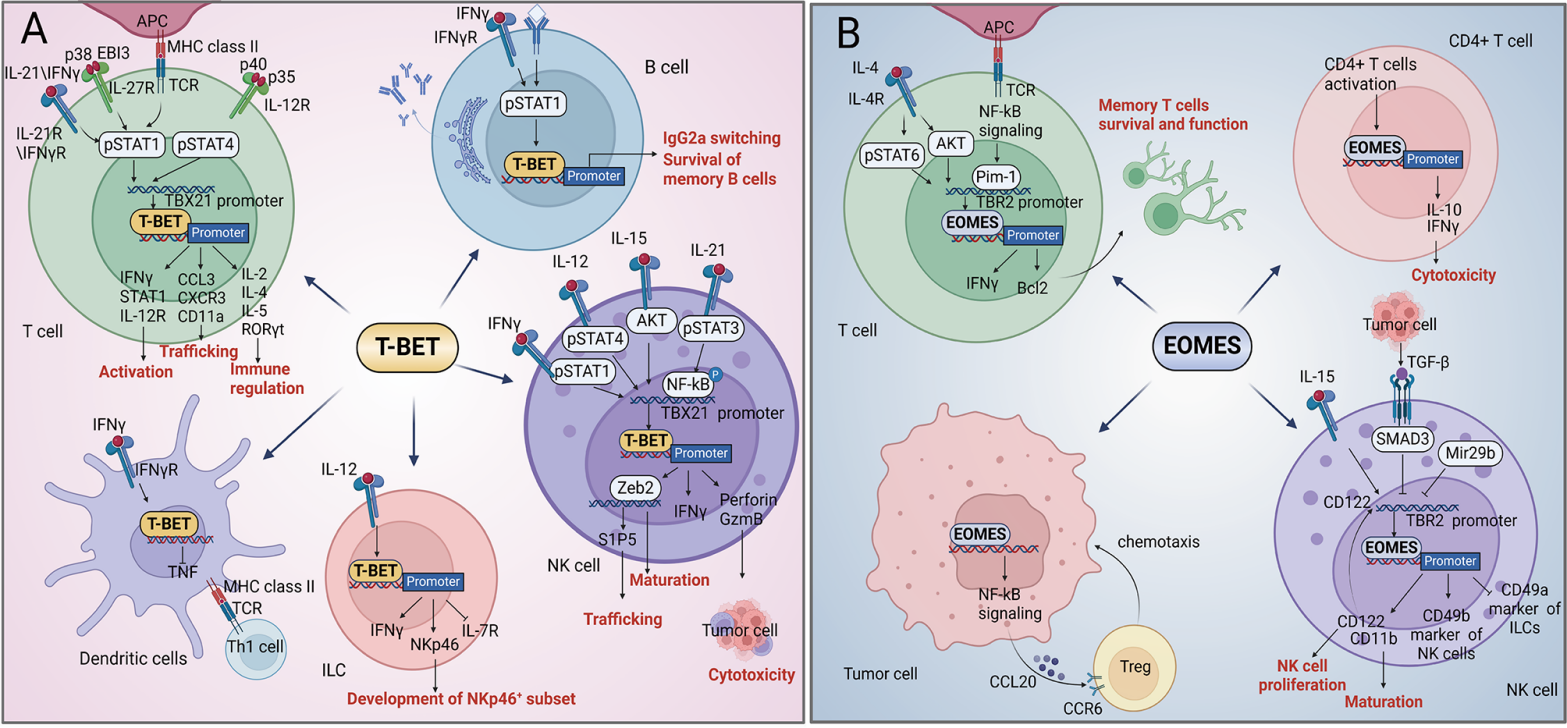
Regulatory mechanisms of T-BET and EOMES in immune microenvironment. **A** T-BET is involved in functioning of various immune cells, including T cells, B cells, DCs, ILC, and NK cells. T cells: T-BET is induced via signaling downstream of TCR and STAT1/4. STAT1 is activated by IFNγR, IFNαR, IL-27R, and IL-21R signaling. STAT4 is activated by IL-12R signaling. T-BET binds to genes that perform different functions in T cells, including activation (IFNγ, STAT1, and IL-12R), cell trafficking (CCL3, CXCR3, and CD11a) and immune regulation (IL-2 and IL-4). B cells: BCR or IFNγR induces T-BET via STAT1, thereby promoting IgG2a conversion and memory B cell survival. NK cells: IFNγ, IL-12, and IL-21 promote T-BET expression via the STAT pathway, and IL-15 activates the PI3K-AKT-mTORC1 signaling axis to regulate T-BET expression. The roles of T-BET in NK cells include promotion of the transcription of genes (perforin and GzmB) for mediation of cytotoxicity, upregulation of IFNγ, and induction of the expression of Zeb2 to promote NK cell maturation. S1P5 regulates the trafficking of NK cells. DCs: IFNγR signaling pathway induces T-BET expression and inhibits TNF expression; proper expression of T-BET activates TH1 cells. ILC: IL-12R signaling induces T-BET expression and promotes development of IFNγ and NKp46-positive subset of cells; **B** EOMES is involved in functioning of various immune cells, including CD4/8^+^ T cells, NK cells, and Treg cells. CD8^+^ T cells: EOMES expression in CD8^+^ T cells is regulated by multiple signaling pathways (TCR, NF-kB, and IL-4R). EOMES promotes IFNγ and BCL expression to maintain survival and function of memory T cells. Treg cells: EOMES affects the chemotaxis of Treg via the NK-kB-CCL20-CCR6 pathway. CD4^+^ T cells: overexpression of EOMES promotes IFNγ and IL-10 production. NK cells: IL-15 induces CD122 expression via upregulation of EOMES, thereby promoting the proliferation of NK cells. In the tumor microenvironment, TGF-β inhibits EOMES expression by binding to SMAD3. EOMES inhibits CD49a (marker of ILCs) expression and induces CD49b (marker of NK cells) expression

Lymphocytes, macrophages, natural killer (NK) cells, and dendritic cells (DCs) are immune cells that have the ability to recognize and destroy neoplastic tumors, limit tumor progression, and protect the host against neoplasm-related attacks. There is considerable evidence that suppressive TIME exists a vital role in tumor initiation, progression, and therapeutic resistance owing to the crosstalk between diverse cell populations via chemokines, cytokines, and immunosuppressive checkpoints [139]. Cell-mediated immune responses against tumors are key processes in tumor immune surveillance [140–142]. Functional immune cells generate tumor-specific antibodies and secrete anti-tumor cytokines, such as NK cells, which destroy tumor cells via direct cytotoxicity [143]. Nearly all research on the function of T-BOX transcription factors in immunity are pertaining to T-BET and EOMES (Fig. 6). T-BET expression is significantly associated with increased Interferon γ (IFNγ) expression in CD4, CD8, and NK cells. T-BET directly activates the *IFNγ* gene by binding to the *IFNγ* promoter and multiple distal regulatory elements located upstream and downstream of the *IFNγ* gene [144]. T-BET-deficient cytotoxic T lymphocytes (CTL) and NK cells exhibit a functional defect. Interestingly, the functional defect is compensated by the expression of EOMES [145], which suggests that the roles of T-BET and EOMES in the immune system are inextricably linke.

### Function of T-BOX factors in T/B cells

**Physiological immunity** T and B cells are important immune cells coordinating various aspects of adaptive immunity, including responses to pathogens, allergens, and tumors. EOMES and T-BET play key roles in T cell activation, differentiation, and memory development, stimulating CD8^+^ T cells to produce potent antiviral activity. T-BET mainly acts on terminally differentiated effector CD8^+^ T cells, whereas EOMES regulates the memory CD8^+^ T cell repertoire [146]. Specifically, T-BET is induced by signaling downstream of the T cell receptor (TCR) and signal transducers and activators of transcription 1 (STAT1), which is activated by interferon α receptor (IFNαR), IFNγR, interleukin-21 receptor (IL-21R), and IL-27R signaling in T cells. T-BET binds a range of genes to perform different functions in T cells including activation (via IFNγ, STAT1, and IL-12R), trafficking (via C-C motif chemokine ligand 3 [CCL3], C-X-C motif chemokine receptor 3 [CXCR3], tyrosine-protein sulfotransferase 2 [TPST2], and CD11a), and immune regulation (via IL-2, IL-4, IL-5, and RAR-related orphan receptor-γ [RORγt]) [147, 148]. EOMES expression in CD8^+^ T cells is controlled by several signaling pathways that are triggered by the activation and differentiation of T cells, including TCR, NF-kB, and IL receptor signaling pathways [149, 150]. The process drives the production of effector cytokines and cytotoxic molecules, such as IFNγ, Bcl-2, perforin, and Granzyme B (GzmB) [150, 151]. T-BET exists a vital role in the function of B cells, particularly in type 1-associated responses. The significant level of T-BET expression in several B cell precursors indicates that it may play a developmental role. Similar to that in T cells, T-BET induces and maintains the type 1-like differentiation program in B cells [145]. IFNγR or B cell receptor (BCR) induces T-BET via STAT1, promoting IgG2a switching and memory B-cell survival [147, 148]. EOMES is expressed at lower levels in CD4^+^ T cells than in CD8^+^ T cells. In CD4^+^ T cells, EOMES overexpression promotes IFNγ production and cytotoxicity. Most of the functions of EOMES observed in CD4^+^ T cells appear to be similar to those of T-BET [152].

**Tumor immunity** Type I immune response involving T helper - type 1 (Th1) cells, γδT cells, CTL, NK cells, and NKT cells has been reported to be a key step of cell-mediated anti-cancer immunity [141]. T-BET expression-deficient T cells show severe impairment of type 1 immunity in mice [145]. T-BET expression in T cells is essential for controlling tumor progression and anti-metastatic activity. Increasing T-BET levels in T-BET-deficient Th1 cells can restore resistance to tumor metastasis [143]. Upregulation of EOMES in CD8^+^ T cells is associated with good clinical response to immunotherapy [153]. For example, enhanced EOMES levels activate CD8^+^ T cells, thereby controlling lymph node metastasis and tumor spread in colorectal cancer [154]. Downregulation of EOMES in tumor-infiltrating T cells and a reduction in EOMES-positive T cells in tumors are poor prognostic factors for HCC [155]. However, EOMES may play a bimodal function in T cells in tumors. EOMES overexpression leads to loss of CD226 in CD8^+^ tumor-infiltrating lymphocytes (TILs), suppression of the function of CD8^+^ T cells, and decrease in the efficacy of cancer immunotherapy [156]. EOMES overexpression also influences the chemotaxis of regulatory T cells (Tregs) via the CC motif chemokine ligand 20 (CCL20)/CC motif chemokine receptor 6 (CCR6) pathway. It causes an immunosuppressive tumor microenvironment to promote ESCA progression [157]. In contrast, the deletion of an *EOMES* allele inhibits the development of depleted CD8^+^ T cells, effectively suppressing tumor progression [158].

### Function of T-BOX factors in NK cells

**Physiological immunity** NK cells are cytotoxic lymphocytes of the innate immune system that kill infectious viruses and cancer cells. T-BET and EOMES drive differentiation and cytotoxic functions of NK cells, and their roles largely overlap [152]; for example, the upregulation of T-BET or EOMES enhances IFNγ production and the killing ability of NK cells; in particular, EOMES-overexpressing NK cells show enhanced antibody-dependent cytotoxicity. EOMES plays a dominant role in the early maturation of NK cells, inducing fast-acting NK receptors and enhancing CD16 expression. In contrast, T-BET regulates explicitly the transcription of terminal maturation markers [159]. Mechanically, molecules such as IFNγ, IL-12, and IL-21 promote T-BET expression via the STAT pathway, and IL-15 activates the PI3K-AKT-mTORC1 signaling axis to regulate T-BET expression. The roles of T-BET in NK cells include.

- Promotion of the transcription of genes such as *PRF1* (encoding perforin) and *GzmB* mediate NK-cell cytotoxicity.
- Upregulation of the secretion of IFNγ regulates the immune response.
- Induction of the expression of Zinc Finger E-Box Binding Homeobox 2 (Zeb2) promotes the differentiation of immature NK cells to mature NK cells.

Zeb2 promotes sphingosine-1-phosphate 5 (S1P5) expression and enables the transfer of mature NK cells from lymph nodes and bone marrow to peripheral organs [152, 160]. IL-15 induces CD122 expression via the upregulation of EOMES, thereby promoting NK cell proliferation; moreover, CD122 is a receptor subunit that maintains NK cell responsiveness to IL-15, thereby creating a positive feedback loop and maintaining normal NK cell development [161].

**Tumor immunity** In the tumor microenvironment, TGF-β downregulates the expression of EOMES by binding to SMAD family member 3 (*SMAD3*). EOMES expression is important for inhibition of CD49a and induction of CD49b, which serve as innate lymphoid cells (ILCs) and NK-cell markers, respectively [152].

### Function of T-BOX factors in DCs

**Physiological immunity** DCs are antigen-presenting cells with a remarkable capacity to activate naïve T cells [162]. T-BET expression in DCs is required for the proper initiation of Th1 cells. T-BET also inhibits TNF production in colonic DCs, which are required to maintain mucosal homeostasis [148].

**Tumor immunity** Interferon-producing killer dendritic cells (IKDCs) produce IFNγ upon tumor recognition. Xu et al. [163] demonstrated that IKDCs overexpressing T-BET (T-BET-IKDCs) showed enhancing cytotoxicity against HCC. An in vivo study conducted using H22-bearing mice confirmed the upregulation of cytotoxic molecules expression, IFNγ secretion increasing, and reduction in tumor size after T-BET-IKDC administration [163]. Thus, T-BET-IKDCs are a potential new immunotherapy for HCC.

Notably, combining multi-omics approaches such as single-cell transcriptome, immunobanking and epigenome, it is possible to map the comprehensive T-BOX family in tumor immunity, systematically depict the temporal heterogeneity, spatial heterogeneity and epigenetic regulatory mechanisms of T-BOX in immune cells, and reveal the potential pathways by which the T-BOX family induces dynamic differentiation of immune cells. A recent study identified extrafollicular pathway-associated B cells characterized by CD21-CD11c and T-BET, defining a new subpopulation of atypical memory (AtM) B cells, which are widely present in the TME and highly correlated with the formation of immunosuppression [164], suggesting the possibility of T-BET as an immunotherapeutic target. In-depth analysis of the effects and regulatory pathways of the T-BOX family on immune cell function in TIME helps to depict tumor-specific patterns and clonal branching. It provides an important reference for subsequent T-BOX studies and a new idea for tumor therapy targeting the T-BOX family in immune cells.

## T-BOX transcription factors in tumor treatment

As regulators of multiple signaling pathways and tumor-associated genes, transcription factors are better biomarkers and potential therapeutic targets than the target genes [40]. Targeting specific transcription factors could provide a potent and effective strategy for fighting cancer, as they usually play a role on upstream of effector cytokines and act on hundreds of related immune-reactive proteins and oncogenes that suppress or promote cancer. T-BOX transcription factors still need to be well-studied and clinically tested as therapeutic targets. With their important role in oncology, T-BOX transcription factors may become key cancer therapeutic targets. However, targeting transcription factors remains a challenging puzzle. A comprehensive understanding of the current therapeutic advances in transcription factors can provide better ideas for targeted therapy.

### TBXT

The selective expression of TBXT in tumor cells and its function in EMT indicates that *TBXT* may be an attractive target for anti-tumor therapy. *TBXT* is chosen as the target of an anti-tumor vaccine because it fulfills two main conditions. The first is tumor specificity, as its expression is almost exclusively restricted to adult tumor tissue. The second feature is immunogenicity [165], revealed by in vitro experiments, which demonstrated that proximity-specific cytotoxic CD8^+^ T cells could be amplified from the blood of patients with cancer [166]. One study indicated that TBXT-specific T cells lyse human breast tumor cells, providing a theoretical basis for the use of a TBXT-specific vaccine as a monotherapy or in combination therapeutic approaches for breast cancer [134]. Two vaccines, MVA-brachyury-TRICOM (NCT02179515) and GI-6301 (NCT01519817), are in Phase I clinical trials to test the safety and efficacy of cancer vaccines targeting Brachyury proteins in pan-cancer cells (lung cancer, breast cancer, PCa, CRC, etc.) [167].

### TBX3

Although *TBX3* has been identified as a novel therapeutic target, directly targeting transcription factors is challenging. A more feasible approach for inhibiting the oncogenic activity of TBX3 is to target its interacting molecules. AS1411 disrupts the synergistic interaction between TBX3 and nucleolin, inhibiting the pro-proliferative and pro-migratory effects of TBX3 in chondrosarcoma, rhabdomyosarcoma, and liposarcoma cell lines [168]. Additionally, miR-137 substitution is a potential therapeutic strategy for melanoma. miR-137 significantly decreases endogenous TBX3 levels and inhibits malignant melanoma cells anchorage-independent growth and migration [169].

### TBX19

In our previous study, *Rac1* and *EGFR* were shown to be direct transcriptional targets of TBX19. We found that using a combination of NSC23766, RAC1 inhibitor, and erlotinib, an EGFR inhibitor, prominently suppressed TBX19-mediated HCC metastasis. This finding provides a promising immunotherapeutic strategy and suggests that TBX19 is a key biomarker for guiding immunotherapy [5].

### T-BET

T-BET expression promotes NK cell function during tumor suppression. Therapies that enhance T-BET expression in NK cells may improve the control of tumor spread [145]. Activation of T-BET or targeted induction of T-BET expression to stimulate IFNγ production in various immune cells holds potential for the treatment of subcutaneous and metastatic melanoma tumors. This strategy could also apply to other tumors and clinical oncology patients [143].

## Conclusions and prospects

The function of the T-BOX family in embryonic, organ, and limb development, craniofacial effects, and other physiological functions is extensively explored in many studies. Recent evidence has revealed a clear relationship between T-BOX members and tumorigenesis, metastasis, proliferation, tumor cell stemness, prognosis, drug resistance, and treatment. The context-dependence of the T-BOX member family is reflected in multiple aspects. In different genders, organs and disease microenvironments, the same T-BOX member plays different or even opposite roles. Mechanistically, different cofactor interactions and genetic modifications lead to the activation of diverse downstream molecules and signaling pathways, resulting in the context-dependence of T-BOX. In recent years, the role of the T-BOX in TIME has begun to receive attention. However, only preliminary studies have been conducted till now; specifically, only T-BET and EOMES have been studied in detail, and the role and the mechanism of action of most T-BOX molecules in the TIME are still unclear. Comprehensive exploration of the T-BOX family in the immune microenvironment is key to subsequently targeting the T-BOX family to improve TIME to enhance therapeutic efficacy. In addition, several clinical trials of T-BOX transcription factors as therapeutic targets or tumor vaccines are ongoing. However, difficulties in using transcription factors as targets for clinical therapies still exists, and many uncharted territories need to be explored. From biology to therapy, the comprehensive exposition of the context-dependent T-BOX family aims to provide new perspectives and insights for using T-BOX in the targeted therapeutic options.

## Data Availability

The datasets generated during the current study are available in GEPIA software (Gene Expression Profiling Interactive Analysis; cancer-pku.cn) and JASPAR (https://jaspar.genereg.net).

## Abbreviations

ABI3BP: ABI family member 3 binding protein
ACC: Adenoid cystic carcinoma
AKAP12: A-Kinase anchoring protein 12
ANF: Atrial natriuretic factor
ARHGAP29: Rho GTPase Activating Protein 29
BAF60a: BRG1-associated factor 60a
BCC: Basal cell carcinoma
BCR: B cell receptor
BCL2L1: BCL2 Like 1
BIRC5: Baculoviral IAP Repeat Containing 5
CA125: Carbohydrate antigen 125
CBFB: Core-Binding Factor Subunit Beta
CBX3: Chromobox3
CC: Cervical cancer
CCL: C-C motif chemokine ligand
CCN4: Cellular Communication Network Factor 4
ccRCC: Clear cell renal cell carcinoma
CDDP: cisplatin
CDKN1C: Cyclin Dependent Kinase Inhibitor 1 C
CDKN2A: Cyclin Dependent Kinase Inhibitor 2 A
CD19^+^: Cluster of differentiation 19^+^
CHDs: Congenital heart defects
circPVT1: CircRNA plasmacytoma variant translocation 1
COL1A1: Collagen type I alpha 1 chain
COL1A2: Collagen Type I Alpha 2 Chain
CRC: Colorectal cancer
CSCs: Cancer stem cells
CST6: Cystatin E/M
CTL: Cytotoxic T lymphocyte
CXCR: C-X-C motif chemokine receptor
DCs: Dendritic cells
DOX: Doxorubicin
EGFR: Epidermal growth factor receptor
EGR1: Early growth response factor 1
EMT: Epithelial-mesenchymal transition
EP300: E1A Binding Protein P300
ERK: Extracellular signal-regulated kinase
ESCA: Esophageal carcinoma
FGF: Fibroblast growth factor
GATA4: GATA binding protein 4
GBM: Glioblastoma
GC: Gastric cancer
GEM: Gemcitabine
GSK3: Glycogen synthase kinase-3
GzmB: Granzyme B
HCC: Hepatocellular carcinoma
HDAC: Histone deacetylase
HER2: Human epidermal growth factor receptor 2
HMGB2: High Mobility Group Box 2
HPV: Human papilloma virus
HP1: Heterochromatin Protein 1
ID1: Inhibitor of DNA binding 1
IFNγ: Interferon-γ
LINC00111: Long Intergenic Non-Protein Coding RNA 111
IKDC: Interferon-producing killer dendritic cell
IKZF2: IKAROS Family Zinc Finger 2
IL: Interleukin
ILCs: Innate lymphoid cells
KAP1: KRAB associated protein 1
KCNH2: Potassium voltage-gated channel subfamily H member 2
KIF2C: Kinesin Family Member 2 C
LEF1: Lymphoid enhancer binding factor 1
MEF2C: Myocyte enhancer factor 2 C
MFF: Mitochondrial Fission Factor
MITF: Microphthalmia-associated transcription factor
MMPs: Matrix metalloproteinases
MTA2: Metastasis Associated 1 Family Member 2
MTSS1: MTSS I-BAR Domain Containing 1
MYH6: Myosin Heavy Chain 6
NHEJ: Non-homologous DNA end-joining
NK cell: Natural killer cell
NKX2.5: NK2 homeobox 5
NCOA5: Nuclear Receptor Coactivator 5
NCOR1/2: Nuclear receptor corepressor 1/2
NDRG1: N-Myc Downstream Regulated 1
NSCLC: Non-small cell lung cancer
PCa: Prostate cancer
PDAC: Pancreatic ductal adenocarcinoma
PDZRN3: PDZ Domain Containing Ring Finger 3
PECAM: Platelet and endothelial cell adhesion molecule 1
PHLPP2: PH domain and leucine-rich repeat protein phosphatase 2
PKC: Protein kinase C
PKM2: Pyruvate kinase M2
PLC: Phospholipase C
PLD1: Phospholipase D1
PTC: Papillary thyroid carcinoma
PTEN: Phosphatase and tensin homolog
PTPRQ: Protein tyrosine phosphatase receptor type Q
PRMT1: Protein arginine methyltransferase 1
p-Rb: phosphorylated Rb
Rac1: Rac family small GTPase 1
RMS: Rhabdomyosarcoma
RORγt: Retinoic acid receptor-related orphan receptor-γt
ROS: Reactive oxygen species
SBNO2: Strawberry Notch Homolog 2
SETD7: SET domain containing lysine methyltransferase 7
SCC: Squamous-cell carcinoma
SFRP1: Secreted frizzled related protein 1
S1P5: Sphingosine-1-phosphate 5
SIRT1: Sirtuin-1
SKCM: Skin cutaneous melanoma
SMAD3: SMAD family member 3
SNCG: Synuclein Gamma
STAT1: Signal transducers and activators of transcription 1
SOX5: SRY-Box transcription factor 5
SP1: Sp1 transcription factor
TCR: T cell receptor
TERT: Telomerase reverse transcriptase
TGF-β: Transforming growth factor-β
THRB: Thyroid hormone receptor beta
Th1: T helper 1 cell
TILs: Tumor-infiltrating lymphocytes
TIME: Tumor immune microenvironment
TNF: Tumor necrosis factor
TPST2: Tyrosine protein sulfotransferase 2
Treg: Regulation T cell
UBF: Upstream Binding Factor
up-oDSGC: upward-preferring ON direction-selective ganglion cell
UTR: Untranslated regions
VCAM1: Vascular cell adhesion molecule 1
YAP1: Yes-associated protein 1
Zeb2: Zinc Finger E-Box Binding Homeobox 2
ZO1: Zona occludens 1
